# Improved method for estimating adlayer thickness and bulk RI change for gold nanocrescent sensors

**DOI:** 10.1038/s41598-018-24950-7

**Published:** 2018-04-27

**Authors:** Ahmed Abumazwed, Wakana Kubo, Takuo Tanaka, Andrew G. Kirk

**Affiliations:** 1 Department of Electrical and Computer Engineering, McGill University, Montreal, H3A 2A9 Canada; 2 Department of Electrical and Electronic Engineering, Tokyo University of Agriculture and Technology, Tokyo, 184-8588 Japan; 3 Metamaterials Laboratory, RIKEN, Wako, Saitama 351-0198 Japan; 4 Innovative Photon Manipulation Research Team, RIKEN Center for Advanced Photonics, Wako, Saitama 351-0198 Japan

## Abstract

This paper presents a novel method employing the maximum likelihood estimation (MLE) technique alongside a nonlinear sensor response model to improve and extract more quantitative sensing results for localized surface plasmon resonance biosensors. The nonlinear response model treats the sensor response as a nonlinear function of the biomolecular adlayer thickness. This method makes use of the multiple resonance characteristic of nanocrescent structures in order to estimate the adlayer thickness and bulk refractive index (RI) change. Nanoimprint lithography is used here to fabricate the nanostructures. The finite element method (FEM) is used to model the nanocrescents and numerically validate the nonlinear-MLE method. Comparing to the established linear model, the proposed nonlinear-MLE method achieves 75% improvement in the limit of detection based on the estimated adlayer thickness and improves the bulk RI resolution by two orders of magnitude.

## Introduction

Localized Surface Plasmon Resonance (LSPR) biosensors have been of interest in sensing applications due to their plasmonic properties, small size and small sample requirement. LSPR sensors have a smaller sensing volume than propagating SPR sensors, hence a higher sensitivity per unit volume based on local changes^1–3^. This is attributed to the reduced interfering effects from the bulk RI changes when sensing small molecules. In addition, SPR sensors require a temperature a controller as the effect of bulk refractive index change significantly contributes to the detected signal. In the case of LSPR sensors, the size of the nanostructures is comparable to the size of the small biological molecules, increasing the specificity and reducing the effect of bulk RI change on the measured quantity (a temperature controller is not needed). We previously introduced the projection method that directly measures the effective refractive index with improved signal to noise ratio (SNR) for LSPR sensors. The effective refractive index is influenced by the adlayer thickness and the change in bulk refractive index^4^. Although the projection method provides a direct measurement for the effective bulk RI change, it cannot be used to distinguish between the effects due to biomolecular adlayer and those due to the bulk RI change, and it was affected by systematic errors and noise. This paper provides a novel method to mitigate these effects and yield quantitative information about binding evens (adlayer thickness and bulk RI change).

Most of surface plasmon resonance (SPR) sensors deploy a reference channel to reduce the interfering effects due to the bulk refractive index (RI) change^5^. However, several methods have been previously proposed for self–referencing SPR sensors, including an integration measurement of reflected intensity from arbitrarily distributed sensing and referencing spots^6^. A self referencing SPR platform was previously introduced based on a microcapillary, where the SPR mode was used as a sensing mode and the Fabry-Perot (FP) mode was used as a ref.^7^. Although the system has employed the low sensitivity of the FP mode to temperature variations, other factors may affect the sensing results, including mass transport. Another approach based on a fiber optic particle plasmon resonance was proposed, employing two plastic-silica fiber optics (with core and cladding diameters of 400 μm and 430 μm, respectively) as sensing and reference channels^8^. The metal nanoparticles were anchored on an unclad sensing fiber of 20 mm sensing window, whereas the other unclad fiber optic was used as a reference channel. This platform was shown to reduce the interfering effects, but it does not provide quantitative information about the binding molecules.

U-shaped nanostructures have been previously used in differentiating between specific and nonspecific binding by extracting information from two or more LSPR modes^9^. The study assumed that specific binding mostly occurs at the metal surface, and the nonspecific binding occurs at a distant location (on the substrate), and ignored any non-specific binding that may occur at the metal surface. However, the method requires repetitive simulation in order to determine the sensitivity matrix based on other biological samples, which is a practical limitation of the method. Alternatively, a model that distinguishes bulk RI and adlayer thickness changes is a more practical solution, as it can decouple the effects associated with them. The sensor response–at each resonance–is related to the adlayer thickness and bulk RI changes,and the effects can be determined by solving the two equations (corresponding to the number of resonances). This linear response model has been previously employed for an SPR sensor with a dielectric overlayer to excite two resonances^10^, an SPR sensor based on simultaneous excitation of short and long range SPR modes^11,12^, a dual-resonance SPR sensor with different penetration depths^13^, simultaneous excitation of transverse and longitudinal modes of nanorods^14^, and a sensor based on surface plasmon resonance and plasmon waveguide resonance^15^. This model, however, requires a sensitivity matrix (including the adlayer and bulk RI sensitivities for both resonances) with a low condition number to avoid any numerical errors, which may not be the case for many sensing platforms. We have improved this method by applying the MLE method to a set of results based on three-resonance nanorod structures, estimating the adlayer thickness and the bulk RI change^16^. Although the accuracy and precision have been improved with self-referencing capabilities, the method remains valid only for extremely low adlayer thickness (<*l*_*d*_/10). This article presents a method based on the maximum likelihood estimation (MLE) technique alongside a nonlinear response model to improve the accuracy and precision of the estimated quantities. This indicates a considerable improvement in the RI resolution and the limit of detection and sensing dynamic range (<*l*_*d*_/2). Herein the proposed method is applied to nanocrescent structures as they can be fabricated using cost-effective fabrication methods and they feature a multiple resonance absorption spectrum^17^. However, the method can be applied to any multiple resonance sensor.

## Nonlinear Model for Sensor Response

**Figure 1:**
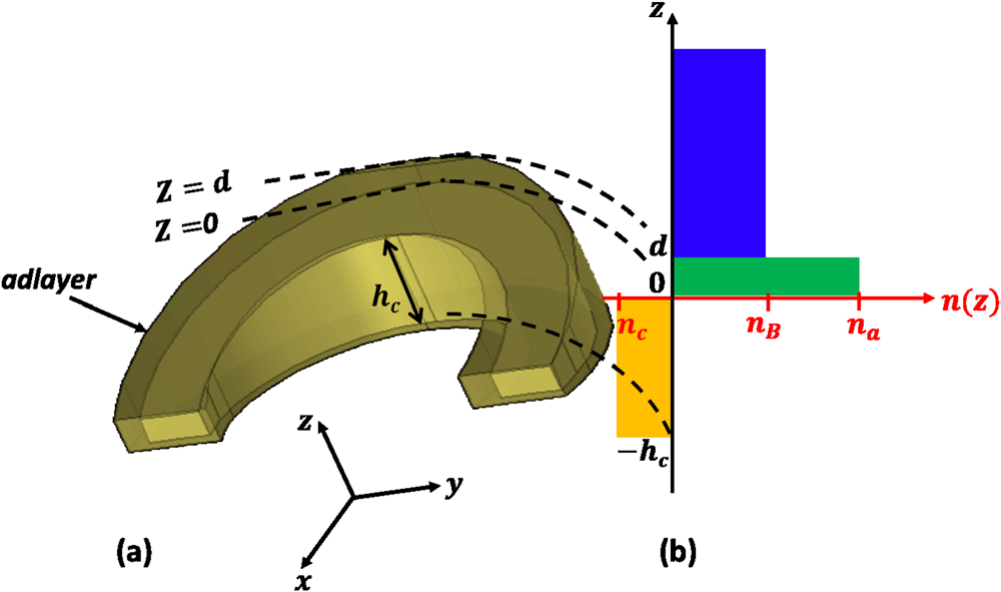
Schematic illustrating the refractive index distribution around a single nanocrescent.

The electromagnetic field decays exponentially from the surface of the nanostructures. If the maximum EM field is located at *z* = 0, it decays with a factor of *exp*(−*z*/*l*_*d*_) along the *z* direction, where *l*_*d*_ is the electromagnetic (EM) decay length, see Fig. 1

Since the intensity is the square of the electric field strength, it decays with a factor of *exp*(−2*z*/*l*_*d*_) with respect to the nanostructure surface (z). This suggests the following for the effective refractive index

1 $${n}_{eff}={n}_{a}\mathrm{[1}-\exp (-2d/{l}_{d})]+{n}_{B}\,\exp (-2d/{l}_{d})$$

2 $$={n}_{B}+({n}_{a}-{n}_{B}\mathrm{)[1}-\exp (-2d/{l}_{d})]$$

Accordingly, the effective refractive index along the z direction is weighted by using the same decay factor *exp*(−2*d*/*l*_*d*_) as shown in Fig. 1. The effective refractive index can be obtained by integrating the refractive indices along z direction as follows^18^

$${n}_{eff}=(2/{l}_{d}){\int }_{0}^{\infty }n(z)\exp (-\,2z/{l}_{d})dz$$

where *n*(*z*) changes along the *z* direction as

3 $$n(z)=(\begin{array}{cc}{n}_{a}, & {\rm{if}}\,0 < z < d,\\ {n}_{B}, & {\rm{if}}\,d < z < \infty \mathrm{.}\end{array}$$

where *n*_*a*_ and *n*_*B*_ are the analyte and bulk refractive indices, respectively. The resonance shift can be determined by

$${\rm{\Delta }}\lambda ={S}_{B}\,({n}_{eff}-{n}_{B})$$

where *S*_*B*_ is the bulk RI sensitivity. From equation (2), substituting for *n*_*eff*_, the following is obtained for the resonance shift

4 $${\rm{\Delta }}\lambda ={S}_{B}\,({n}_{eff}-{n}_{B})\equiv {S}_{B}\,({n}_{a}-{n}_{B}\mathrm{)[1}-\exp (-2d/{l}_{d})]$$

This can be used to estimate the the adlayer thickness *d* and bulk RI change Δ*n*_*B*_. First, the bulk RI sensitivity *S*_*B*_ is measured based on bulk RI change for ethanol solutions of known refractive indices. The nanocrescents support three resonances, but only two resonances are used here. The base solution is injected to obtain the sensing baselines for these resonances, and the shifts in the resonance wavelengths Δ*λ*_*i*_ are tracked in real time as the biological sample is introduced.

The maximum response can be obtained when a thick adsorbate layer is reached *d* → ∞, and equation (4) becomes

5 $${\rm{\Delta }}{\lambda }_{max}={S}_{B}({n}_{a}-{n}_{B})$$

Dividing equation (4) by equation (5), the following is obtained

6 $$\frac{{{\rm{\Delta }}}_{\lambda }}{{\rm{\Delta }}{\lambda }_{max}}=\mathrm{[1}-\exp (-2d/{l}_{d})]$$

Equation (6) can be rearranged in order to obtain an expression that is linear in adlayer thickness as follows

7 $$\mathrm{ln}(1-\frac{{\rm{\Delta }}\lambda }{{\rm{\Delta }}{\lambda }_{max}})=\frac{-2}{{l}_{d}}\,d$$

For a nanostructure with multiple resonances, the electromagnetic decay length is dependent on the resonance wavelength, and the bulk RI sensitivity. Considering a two-resonance system and including the errors associated with the measured data, equation (7) can be written as follows

8 $${y}_{i}={C}_{{d}_{i}}\,d\,\pm {\varepsilon }_{{y}_{i}}$$

where *y*_*i*_ = *ln*(1−Δ*λ*_*i*_/Δ*λ*_*i*,*max*_), ${C}_{{d}_{i}}$ is a coefficient related to the electromagnetic field decay length for the *i*^*th*^ resonance calculated as $-2/{l}_{{d}_{i}}$, and ${\varepsilon }_{{y}_{i}}$ represents the error due to noise which is directly related to the variance of the measured quantities ***R***_***y***_ over the measured shifts in resonance wavelengths. In matrix notation

9 $${\boldsymbol{y}}=[\begin{array}{c}{y}_{1}\\ {y}_{2}\end{array}],\,{{\boldsymbol{C}}}_{{\boldsymbol{d}}}=[\begin{array}{c}{C}_{d1}\\ {C}_{d2}\end{array}],\,and\,{{\boldsymbol{R}}}_{{\boldsymbol{y}}}=[\begin{array}{cc}{R}_{y11} & {R}_{y12}\\ {R}_{y12} & {R}_{y22}\end{array}]$$

where *R*_12_ represents the covariance accounting for correlated noise. Considering a multiple resonance response, *y*_1_, *y*_2_, ..., *y*_*i*_, and a normal distribution for the noise effect, the likelihood of obtaining these measured quantities (*y*_*i*_) given the true value ($\hat{d}$) and the variance **R**_***y***_ can be obtained by multiplying the normal distributions ${\mathscr{N}}$ of these estimates. In matrix notation this can be expressed as follows

10 $$P({y}_{1},\mathrm{...}{y}_{i}|{C}_{d1}\hat{d},\mathrm{...}{C}_{di}\,\hat{d},\,{{\boldsymbol{R}}}_{{\boldsymbol{y}}})=\prod _{i}{\mathscr{N}}({\boldsymbol{y}}|{{\boldsymbol{C}}}_{{\bf{d}}}\,\hat{d},{{\boldsymbol{R}}}_{{\boldsymbol{y}}})\equiv \frac{1}{{\mathrm{(2}\pi )}^{i\mathrm{/2}}|{{\boldsymbol{R}}}_{{\boldsymbol{y}}}{|}^{\mathrm{1/2}}}\exp [-\frac{1}{2}{({\boldsymbol{y}}-{{\boldsymbol{C}}}_{{\bf{d}}}\hat{d})}^{T}{{\boldsymbol{R}}}_{{\bf{y}}}^{-{\bf{1}}}({\boldsymbol{y}}-{{\boldsymbol{C}}}_{{\boldsymbol{d}}}\,\hat{d})]$$

According to the MLE, the maximum likelihood of estimating the true value is obtained when the derivative of the above likelihood with respect to the true value approaches zero^19^. For simplicity, we obtain the log of the above likelihoods as follows

11 $$ln\,P({\boldsymbol{y}}|{{\boldsymbol{C}}}_{{\boldsymbol{d}}}\hat{d},\,{{\boldsymbol{R}}}_{{\boldsymbol{y}}})=-\frac{i}{2}ln\mathrm{(2}\pi )-\frac{1}{2}ln|{{\boldsymbol{R}}}_{{\boldsymbol{y}}}|-\frac{1}{2}{({\boldsymbol{y}}-{{\boldsymbol{C}}}_{{\boldsymbol{d}}}\hat{d})}^{T}{{\boldsymbol{R}}}_{{\boldsymbol{y}}}^{-{\bf{1}}}({\boldsymbol{y}}-{{\boldsymbol{C}}}_{{\boldsymbol{d}}}\hat{d})$$

Now, the true value can be estimated such that the derivative of the log likelihood with respect to this true value is equal to zero^19^.

$$\begin{array}{rcl}\frac{\partial }{\partial \hat{d}}\{\,ln\,P({\boldsymbol{y}}|{{\boldsymbol{C}}}_{{\boldsymbol{d}}}\hat{d},{{\boldsymbol{R}}}_{{\bf{y}}})\} & \equiv  & \frac{\partial }{\partial \hat{d}}({{\boldsymbol{y}}}^{{\boldsymbol{T}}}{{\boldsymbol{R}}}_{{\boldsymbol{y}}}^{-{\bf{1}}}{\boldsymbol{y}}-{{\boldsymbol{y}}}^{{\boldsymbol{T}}}{{\boldsymbol{R}}}_{{\boldsymbol{y}}}^{-{\bf{1}}}{{\boldsymbol{C}}}_{{\bf{d}}}\hat{d}-{{\boldsymbol{C}}}_{{\boldsymbol{d}}}^{{\boldsymbol{T}}}{\hat{d}}^{T}{{\boldsymbol{R}}}_{{\boldsymbol{y}}}^{-{\bf{1}}}{\boldsymbol{y}}+{{\boldsymbol{C}}}_{{\boldsymbol{d}}}{\hat{d}}^{T}{{\boldsymbol{R}}}_{{\boldsymbol{y}}}^{-{\bf{1}}}{{\boldsymbol{C}}}_{{\boldsymbol{d}}}\hat{d})\\  & = & 0\Rightarrow -{{\boldsymbol{y}}}^{{\boldsymbol{T}}}{{\boldsymbol{R}}}_{{\boldsymbol{y}}}^{-{\bf{1}}}{{\boldsymbol{C}}}_{{\boldsymbol{d}}}-{{\boldsymbol{C}}}_{{\boldsymbol{d}}}^{{\boldsymbol{T}}}{{\boldsymbol{R}}}_{{\boldsymbol{y}}}^{-{\bf{1}}}{\boldsymbol{y}}+2\,{{\boldsymbol{C}}}_{{\boldsymbol{d}}}^{{\boldsymbol{T}}}{{\boldsymbol{R}}}_{{\boldsymbol{y}}}^{-{\bf{1}}}{{\boldsymbol{C}}}_{{\boldsymbol{d}}}\hat{d}=0\end{array}$$

This can be solved for the estimate $\hat{d}$

12 $$\hat{d}={({{\boldsymbol{C}}}_{{\boldsymbol{d}}}^{{\boldsymbol{T}}}{{\boldsymbol{R}}}_{{\boldsymbol{y}}}^{-{\bf{1}}}{{\boldsymbol{C}}}_{{\boldsymbol{d}}})}^{-1}({{\boldsymbol{C}}}_{{\boldsymbol{d}}}^{{\boldsymbol{T}}}{{\boldsymbol{R}}}_{{\boldsymbol{y}}}^{-{\bf{1}}}{\boldsymbol{y}})$$

In the case of the dual-resonance nanocrescents (*i* = 2), the adlayer thickness can be estimated as follows

13 $$\widehat{d}=\frac{({C}_{d1}{R}_{y11}^{-1}+{C}_{d21}{R}_{y12}^{-1}){y}_{1}+({C}_{d2}{R}_{y22}^{-1}+{C}_{d1}{R}_{y12}^{-1}){y}_{2}}{{C}_{d1}^{2}{R}_{y11}^{-1}+{C}_{d2}^{2}{R}_{y22}^{-1}+2\,{C}_{d1}\,{C}_{d2}{R}_{y12}^{-1}}$$

If the noise is uncorrelated (*R*_*y*12_ = 0), the estimate becomes

14 $$\widehat{d}=\frac{({C}_{d1}{R}_{y11}^{-1}){y}_{1}+({C}_{d2}{R}_{y22}^{-1}){y}_{2}}{{C}_{d1}^{2}{R}_{y11}^{-1}+{C}_{d2}^{2}{R}_{y22}^{-1}}$$

Likewise, the RI change can be estimated with an improved accuracy using the MLE method. From equation (4), the shifts in the resonance wavelengths are given by

$${\rm{\Delta }}{\lambda }_{i}={C}_{{n}_{i}}\,{\rm{\Delta }}n\pm {\varepsilon }_{{\lambda }_{i}}$$

where Δ*n* = *n*_*a*_ − *n*_*B*_±Δ*n*_*B*_, and ${C}_{ni}={S}_{i}(1-{e}^{-2\hat{d}/{l}_{di}})$ represents the sensitivity coefficient for the *i*^*th*^ resonance, and $\hat{d}$ is the value estimated by equation (13) or (14). ${\varepsilon }_{{\lambda }_{i}}$ is the error due to noise, represented by the variance associated with the *i*^*th*^ measured wavelength shift. The estimated $\widehat{{\rm{\Delta }}n}$ that maximizes the likelihood of the above probability assuming a correlated noise can be obtained using the MLE

15 $$\widehat{{\rm{\Delta }}n}=\frac{({C}_{n1}{R}_{{\lambda }_{1}}^{-1}+{C}_{n2}{R}_{{\lambda }_{12}}^{-1}){{\rm{\Delta }}}_{{\lambda }_{1}}+({C}_{n2}{R}_{{\lambda }_{2}}^{-1}+{C}_{n1}{R}_{{\lambda }_{12}}^{-1}){\rm{\Delta }}{\lambda }_{2}}{{C}_{n1}^{2}{R}_{{\lambda }_{1}}^{-1}+{C}_{n2}^{2}{R}_{{\lambda }_{2}}^{-1}+2\,{C}_{n1}\,{C}_{n2}{R}_{{\lambda }_{12}}^{-1}}$$

For an uncorrelated system (${R}_{{\lambda }_{12}}\mathrm{=0}$), this simplifies the above equation to

16 $$\widehat{{\rm{\Delta }}n}=\frac{{C}_{n1}{R}_{{\lambda }_{11}}^{-1}{\rm{\Delta }}{\lambda }_{1}+{C}_{n2}{R}_{{\lambda }_{22}}^{-1}{\rm{\Delta }}{\lambda }_{2}}{{C}_{n1}^{2}{R}_{{\lambda }_{11}}^{-1}+{C}_{n2}^{2}{R}_{{\lambda }_{22}}^{-1}}$$

## Simulated Results and Validation of the Estimation Method

This section compares the nonlinear–MLE method with the established linear response model based on FEM simulated results, and presents a FEM evaluation for the accuracy based on each method with respect to deviated resonance wavelengths (simulating the effect of noise on each resonance wavelength).

The simulated results show distinct values for the sensitivity to bulk RI and adlayer thickness changes. Inspecting both modes in terms of the adlayer and bulk RI sensitivities can provide an insight into the sensing efficiency for each mode. It is evident that the adlayer thickness, the adlayer refractive index *n*_*a*_ and the bulk refractive index *n*_*B*_ contribute to the effective refractive index *n*_*eff*_ [Equation (1)]. Therefore, the adlayer sensitivity (*S*_*d*_) can be given by^20^

17 $$\frac{\partial \lambda }{\partial d}=\frac{\partial \lambda }{\partial {n}_{eff}}\,\frac{\partial {n}_{eff}}{\partial d}$$

where ∂*λ*/∂*d* and ∂*λ*/∂*n*_*eff*_ are the adlayer sensitivity *S*_*d*_ and bulk RI sensitivity *S*_*B*_, respectively. This equation suggests that the adlayer sensitivity is determined by the bulk RI sensitivity and the rate at which the adlayer thickness contributes to the effective refractive index *n*_*eff*_. Using equation (17) we can define the adlayer sensing efficiency *η* of a given biosensor as follows

18 $$\eta =\frac{\partial {n}_{eff}}{\partial d}\equiv \frac{{S}_{d}}{{S}_{B}}$$

**Figure 2:**
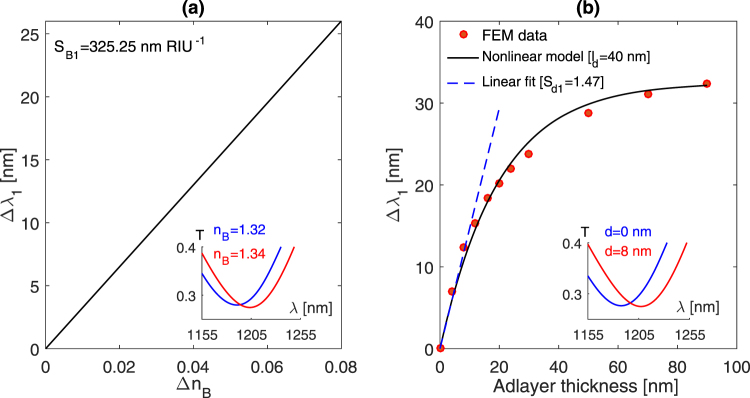
(**a**) Resonance shift versus bulk RI change associated with the first mode supported by exciting the nanocrescent structures with a normal incident, horizontally polarized, plane wave. (**b**) the simulated sensor response due to increasing the adlayer thickness. The results from the nonlinear model are shown on the graph. The FEM model used the following parameters: *n*_*B*_ = 1.3, *n*_*a*_ = 1.4. This mode is located at 1100 nm when *n*_*B*_ = *n*_*a*_ = 1.

**Figure 3:**
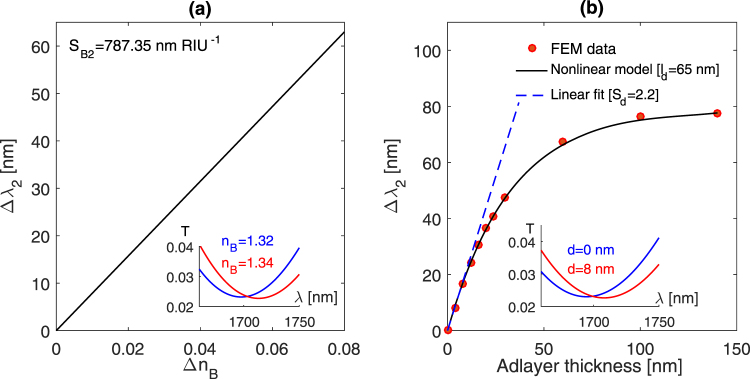
(**a**) Resonance wavelength shift versus bulk RI change associated with the second mode. (**b**) The simulated sensor response due to changing the adlayer thickness. The results from the nonlinear model are shown on the graph. The mode is located at 1450 nm in air (*n*_*B*_ = *n*_*a*_ = 1).

This determines how effectively a given biosensor can detect changes in adlayer thickness. Referring to Figs 2 and 3, the first mode (1200 nm) exhibits a lower bulk RI sensitivity, comparing to that associated with the second mode ~1700 nm (325.25 nm/RIU versus 787.35 nm/RIU). These modes yield adlayer sensitivities of 1.47 and 2.2, respectively. Therefore, the calculated adlayer sensing efficiency *η* is 4.5 × 10^−3^ RIU/nm and 2.8 × 10^−3^ RIU/nm. In other words, changing the adlayer thickness by 1 nm would alter the effective refractive index by 4.5 × 10^−3^ RIU based on the first mode and 2.8 × 10^−3^ RIU in the case of the second mode, although the second mode features a higher bulk RI sensitivity than that of the first mode. We can now consider the established linear response model for comparison, the LM method relates each resonance wavelength shift to the changes in RI and thickness of an adsorbed biological material by the following relationship

19 $$[\begin{array}{c}{\rm{\Delta }}{\lambda }_{1}\\ {\rm{\Delta }}{\lambda }_{2}\end{array}]=\mathop{\underbrace{[\begin{array}{cc}{S}_{{B}_{1}} & {S}_{{d}_{1}}\\ {S}_{{B}_{2}} & {S}_{{d}_{2}}\end{array}]}}\limits_{{\bf{S}}}[\begin{array}{c}{\rm{\Delta }}n\\ d\end{array}]$$

The application of the linear model is valid only if: (i) the sensitivity matrix (***S***) is not singular, and (ii) the normalized sensitivity matrix (***S′***) is well conditioned^21^. The normalized sensitivity matrix is calculated as follows

20 $${\boldsymbol{S}}^{\prime} =[\begin{array}{cc}\frac{{S}_{{B}_{1}}}{\sqrt{{S}_{{B}_{1}}^{2}+{S}_{{B}_{2}}^{2}}} & \frac{{S}_{{d}_{1}}}{\sqrt{{S}_{{d}_{1}}^{2}+{S}_{{d}_{2}}^{2}}}\\ \frac{{S}_{{B}_{2}}}{\sqrt{{S}_{{B}_{1}}^{2}+{S}_{{B}_{2}}^{2}}} & \frac{{S}_{{d}_{2}}}{\sqrt{{S}_{{d}_{1}}^{2}+{S}_{{d}_{2}}^{2}}}\end{array}]$$

This matrix meets the above conditions if it has a non-zero determinant and low condition number.

$${\boldsymbol{S}}=\mathop{\underbrace{[\begin{array}{cc}325.25 & 1.02\\ 787.38 & 1.51\end{array}]}}\limits_{|{\boldsymbol{S}}|=-\,366.25\ne 0}\Rightarrow {\boldsymbol{S}}^{\prime} =\mathop{\underbrace{[\begin{array}{cc}0.38 & 0.55\\ 0.92 & 0.84\end{array}]}}\limits_{\kappa ({\boldsymbol{S}}^{\prime} \mathrm{)=10.49 < 100}}$$

From equation (19), the adlayer thickness and bulk RI change can be calculated as

21 $$[\begin{array}{c}{\rm{\Delta }}n\\ d\end{array}]={{\boldsymbol{S}}}^{-{\bf{1}}}[\begin{array}{c}{\rm{\Delta }}{\lambda }_{1}\\ {\rm{\Delta }}{\lambda }_{2}\end{array}]$$

**Figure 4:**
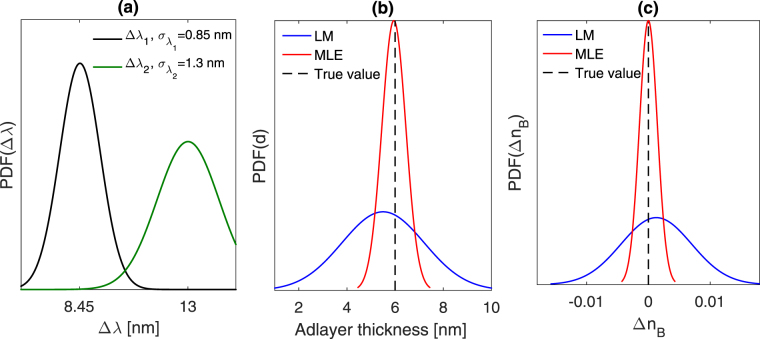
(**a**) Probability density function representation of the calculated shift in the first and second resonances, with a 10 dB signal to noise ratio (SNR = ${\rm{\Delta }}{\lambda }_{i}/{\sigma }_{{\lambda }_{i}}\mathrm{=10}$) [${\sigma }_{{\lambda }_{1}}\mathrm{=0.85}$ nm, ${\sigma }_{{\lambda }_{2}}\mathrm{=1.3}$ nm]. The following parameters were used in the simulation: *n*_*B*_ = 1.33, *n*_*a*_ = 1.43, d = 6 nm. (**b**) The estimated adlayer thickness, and (**c**) the estimated bulk RI change based on the linear model (blue) and the MLE method (red). The precision for the MLE results: *σ*_*d*_ = 0.09 nm, *σ*_*n*_ = 1.6  × 10^−4^ RIU. The precision of the LM: *σ*_*d*_ = 0.25 *nm*, *σ*_*n*_ = 6.14 × 10^−4^ *RIU*.

The nonlinear–MLE method is now employed to estimate the bulk RI change and adlayer thickness–that were used in the simulation–by adding uncertainties to the simulated shifts of the resonance wavelengths ($\pm {\sigma }_{{\lambda }_{i}}$) such as ${\rm{\Delta }}{\lambda }_{i}/{\sigma }_{{\lambda }_{i}}\mathrm{=10}$, as shown in Fig. 4(a). The resonance wavelength shifts are used to determine the corresponding values for *ln*(1−Δ*λ*/Δ*λ*_*max*_), which are then used in equation (14) to determine the adlayer thickness, as shown in Fig. 4(b). The adlayer thickness is then used to determine the sensitivity coefficient, *C*_*n*_ = *S*[1−*exp*(−2*d*/*l*_*d*_)], to estimate the bulk RI change using equation (16). The estimated adlayer thickness and bulk RI change are shown in Fig. 4(c). The results obtained based on the LM are also shown in Fig. 4(b,c). The nonlinear-MLE method revealed the following for the estimates: d = 5.95 nm, Δ*n*_*B*_ ≈ 0, with 0.47 nm and 1.4 × 10^–3^ RIU uncertainties, respectively. Under the same conditions, the linear response model revealed 5.5 nm and 1.3 × 10^−3^ for the estimated adlayer thickness and bulk RI change with uncertainties of 1.8 nm and 6 × 10^−3^, respectively. This suggests that the nonlinear–MLE can improve the accuracy of the estimated adlayer thickness by one order of magnitude, and the precision by a factor of 4, as shown in Fig. 4.

**Figure 5:**
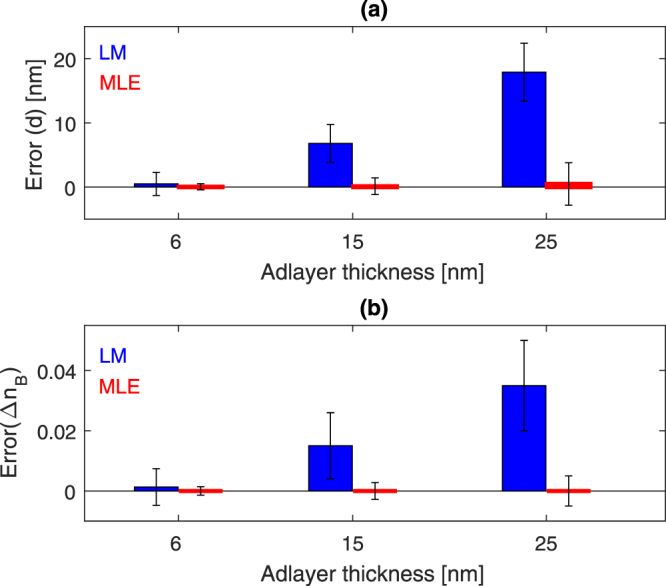
(**a**) Error in the estimated adlayer thickness based on the nonlinear–MLE method (red bars) and the linear response model (blue bars). The following parameters were used in the FEM model: *n*_*a*_ = 1.43, *n*_*B*_ = 1.33, and *d* = {6 *nm*, 15 *nm*, 25 *nm*}. The shifts in the resonance wavelengths were determined, and each resonance was added uncertainty ${\sigma }_{{\lambda }_{i}}$, such that each ${\rm{\Delta }}{\lambda }_{i}/{\sigma }_{{\lambda }_{i}}\mathrm{=10}$ (SNR = 10). The error was then determined as the difference between the true and estimated values. (**b**) The error associated with estimated Δ*n* based on the same simulated results (resonance wavelength shifts) used in (**a**).

Figure 5 compares both methods in terms of accuracy and precision based on adlayer thickness ranging from 6 nm to 25 nm. The percentage error in the estimated adlayer thickness ranges from 0.83–1.96% based on the nonlinear–MLE method, and 8.3–71.6%, based on the linear response model. This indicates that the nonlinear–MLE method can improve the results by a factor of 36 as compared to those based on the linear response model, when the adlayer thickness approaches $\sim {l}_{d}/2$. The error in the estimated RI change tends to be negligible based on the nonlinear–MLE method and increases drastically based on the linear response model, as shown in Fig. 5(b). The nonlinear–MLE method and the linear response model reveal 5 × 10^−3^ and 1.5 × 10^−2^ RIU uncertainties when the adlayer thickness approaches 25 nm, as shown Fig. 5(b).

## Experimental Results

This section provides the experimental results based on the Bioten-Streptavidin binding events. The samples were prepared according to the established protocol^9,22^. Before being used in the sensing experiments, the fabricated nanocrescent structures were cleaned by DI water and ethanol solution, blown dry with nitrogen,and plasma treated to remove any biological contaminant. The substrate was then functionalized by biotin-hpdp, and streptavidin solution was prepared in tris-buffer according to the established surface chemistry protocol^9,22^. The bulk RI sensitivity at each resonance was first determined based on resonance shift against RI change due to changing the concentration in ethanol solution. The sensitivities to adlayer thickness were then determined based on the measured results by correcting the simulated counterparts based on the measured bulk RI sensitivities. Since the adayer sensitivity is related to the bulk RI sensitivity by *S*_*d*_ = *S*_*B*_(*n*_*a*_−*n*_*B*_)/*l*_*d*_. The corrected adlayer sensitivity ${S}_{d}^{{\prime} }$ can be determined as follows

22 $${S}_{d}^{{\prime} }={S}_{d}\,S{^{\prime} }_{B}/{S}_{B}$$

where *S*_*d*_ is the simulated adlayer sensitivity and *S*_*B*_ and ${S}_{B}^{{\prime} }$ are the simulated and measured buk RI sensitivities, respectively. The bulk RI sensitivity is used along the EM decay length in equations (14) and (16) to estimate the adlayer thickness and bulk RI change, respectively, based on the nonlinear–MLE method. This section also compares these results to those obtained based on the linear model. The linear model uses the bulk and adlayer sensitivity factors in equation (19) to estimate the adlayer thickness and bulk RI change.

**Figure 6:**
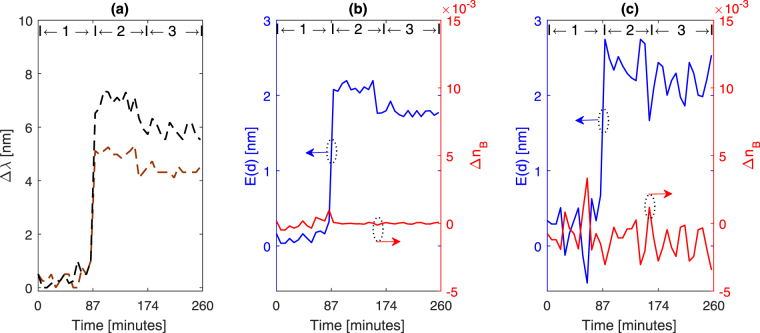
(**a**) Shifts in resonance wavelengths (*λ*_1_ = 1200 *nm*, *λ*_2_ = 1700 *nm*) with respect to streptavidin-bioten binding. (**b**) Adlayer thickness (left y-axis) and bulk RI change (right y-axis) estimated by the nonlinear-MLE method. (**c**) Estimated adlayer thickness (left y-axis) and the bulk RI change (right y-axis) based on the linear response model. The sequence on the graphs indicates the order of introducing the solutions to the nanocrescents [1]: Tris buffer solution [2], streptavidin, and [3] Tris buffer solution.

Figure 6(a) shows the shifts in the resonance wavelengths, based of the measured extinction curves for streptavidin biotin sensing experiments. These shifts were translated into the logarithmic normalized quantity, *ln*(1 − Δ*λ*/Δ*λ*_*max*_), and used in equations (14) and equation (16) to estimate the adlayer thickness and bulk RI change, as shown in Fig. 6(b,c). Now the linear model is used based on the same measured data, shown in Fig. 6(a) in estimating the adlayer thickness and bulk RI change using the sensitivity factors in equation (19). Figure 6(c) shows the results based on the linear model. The limit of detection and bulk RI resolution can be determined based on the standard deviation of the estimated adlayer thickness and bulk RI change, respectively. Based on these results, the nonlinear-MLE method improves the accuracy and precision as compared with the linear model. The limit of detection for adlayer thickness is reduced by a factor of 4, and the bulk RI resolution is improved by a factor of 22 based on the bulk RI change. Table 1 compares between the proposed nonlinear-MLE method and the linear model based on the estimated adlayer thickness and measured sensing characteristics.

**Table 1:** Estimated adlayer thickness and sensing characteristics based on the nonlinear-MLE method and the linear model.

	Adlayer thickness [nm]	${{\boldsymbol{\sigma }}}_{\hat{{\bf{d}}}}$ [nm]	*σ*_*n*_ [RIU]
LM	2.16	0.24	1.3×10^−3^
Nonlinear–MLE	1.78	0.06	6×10^−5^

## Discussion

The paper presented a statistical method, combining the the MLE method with a nonlinear model, for extracting the adlayer thickness and bulk RI change with improved accuracy. The method not only provided a quantitative information about the binding events, but also improved the precision of LSPR sensors. The paper adopted a nonlinear model for the sensor response, to adlayer thickness change, which reflects the real scenario of typical sensing experiments. The model follows a similar trend to that based on the association constant in chemical sensors.

The nonlinear model uses the EM field decay length and sensitivity for each resonance to estimate the adlayer and bulk RI change, whilst the linear model uses the sensitivity to bulk RI change and the adlayer sensitivity that needs to be recalculated (corrected) for other target biomolecules. The latter represents a substantial disadvantage of the linear model. In contrast to the linear model, the nonlinear model avoids the redundant calculation of the sensitivity to adlayer thickness for various biomolecular adlayer. Based on the simulated and measured result, and compared to the linear model, the nonlinear–MLE method improved the bulk RI resolution by two orders of magnitude (6 × 10^−5^ RIU vs 1.3 × 10^−3^ RIU). The method also achieves 75% improvement in the limit of detection based on the adlayer thickness (0.06 nm vs 0.24 nm uncertainty in the estimated adlayer thickness). In this paper, we considered the nanocrescent structures, but the proposed method can be applied to a wide range of different structures and various sensing scenarios.

## Methods

**Figure 7:**
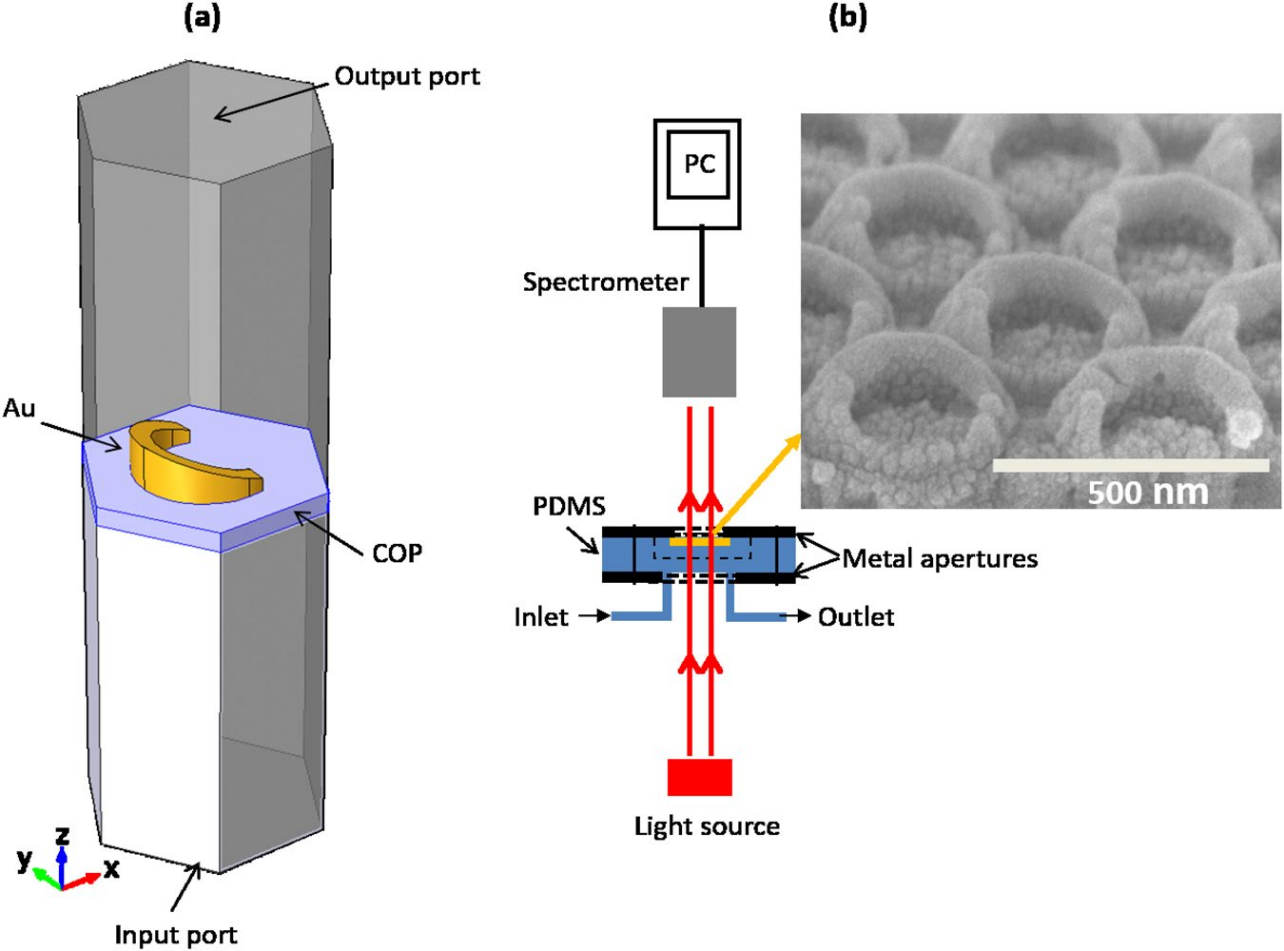
(**a**) Simulation layout used in COMSOL Multiphysics to model periodic nanocrescent structure of a hexagonal lattice. (**b**) Experimental set-up to measure the transmission spectra for the fabricated structure. The inset shows a SEM image for the fabricated nanocrescents (50 nm in thickness and 90 nm in height with 20° wedge angle).

The finite element method (FEM) was used to calculate the sensor response to bulk RI and adlayer thickness changes and validate the MLE method based on the nonlinear model. Periodic boundary conditions were enforced along the sides of a hexagonal simulation domain created with commercial COMSOL Multiphysics simulation package, as shown in Fig. 7(a). The refractive index for gold was obtained from Johnson and Christy^23^.The nanocrescents and the adlayer were discretized using a tetrahedral mesh, and triangular mesh was used with the rest of the domain. The transmission efficiency was obtained from the scattering parameter *S*_21_, as input and output ports were assigned to the bottom and top surfaces of the simulation domain, shown in Fig. 7(a).

The nanostructures were fabricated on a cyclic olefin polymer (COP) substrate by the nanoimprint lithography method and reactive ion etching as explained in a previous work^17^. A scanning electron microscope (SEM) image for the fabricated structures is shown in the inset of Fig. 7(b).

The fluidic channel used in the experiments was fabricated using replica moulding method as explained in^16,24^. Cary 5000 spectrometer was used in the set-up, illustrated in Fig. 7(b), to measure the extinction curves for the nanocrescents while injecting the ethanol and streptavidin solutions.

## References

[1] Willets KA, Van Duyne RP (2007). Localized surface plasmon resonance spectroscopy and sensing. Annu. Rev. Phys. Chem..

[2] Anker JN (2008). Biosensing with plasmonic nanosensors. Nature Mater..

[3] Bingham JM, Anker JN, Kreno LE, Van Duyne RP (2010). Gas sensing with high-resolution localized surface plasmon resonance spectroscopy. J. Am. Chem. Soc..

[4] Abumazwed A, Kubo W, Shen C, Tanaka T, Kirk AG (2016). Projection method for improving signal to noise ratio of localized surface plasmon resonance biosensors. Biomed. Opt. Express.

[5] O’Brien I (1999). SPR biosensors: simultaneously removing thermal and bulk-composition effects. Biosens.Bioelectron..

[6] Nizamov S, Scherbahn V, Mirsky VM (2015). Self-referencing SPR-sensor based on integral measurements of light intensity reflected by arbitrarily distributed sensing and referencing spots. Sensors and Actuators B: Chemical.

[7] Chen S, Liu Y, Liu Z, Chu S, Peng W (2016). Micro-capillary-based self-referencing surface plasmon resonance biosensor for determination of transferrin. Appl. Opt..

[8] Wu C-W (2016). Self-referencing fiber optic particle plasmon resonance sensing system for real-time biological monitoring. Talanta.

[9] Nehru N, Linliang Y, Yinan W, Hastings JT (2014). Using U-shaped localized surface plasmon resonance sensors to compensate for nonspecific interactions. IEEE Trans. Nanotechnology.

[10] Homola J, Lu HB, Yee SS (1999). Dual-channel surface plasmon resonance sensor with spectral discrimination of sensing channels using dielectric overlayer. Electronics Letters.

[11] Slavik, R., Homola, J. & Vaisocherová, H. Advanced biosensing using simultaneous excitation of short and long range surface plasmons. *Measurement Science and Technology* **17** (2006).

[12] Hastings J (2007). Optimal self-referenced sensing using long- and short- range surface plasmons. Opt. Express.

[13] Nizamov S, Mirsky VM (2011). Self-referencing SPR-biosensors based on penetration difference of evanescent waves. Biosens. Bioelectron..

[14] Nehru N (2012). Differentiating surface and bulk interactions using localized surface plasmon resonances of gold nanorods. Opt. Express.

[15] Bahrami F, Maisonneuve M, Meunier M, Aitchison JS, Mojahedi M (2014). Self-referenced spectroscopy using plasmon waveguide resonance biosensor. Biomed. Opt. Express.

[16] Abumazwed A, Kubo W, Tanaka T, Kirk AG (2017). Improved self-referenced biosensing with emphasis on multiple-resonance nanorod sensors. Opt. Express.

[17] Kubo W, Fujikawa S (2011). Au double nanopillars with nanogap for plasmonic sensor. Nano Letters.

[18] Jung LS, Campbell CT, Chinowsky TM, Mar MN, Yee SS (1998). Quantitative interpretation of the response of surface plasmon resonance sensors to adsorbed films. Langmuir.

[19] Scharf, L. L. Statistical Signal Processing: Detection, Estimation, and Time Series Analysis (Addison-Wesley, 1991).

[20] Piliarik M, Homola J (2009). Surface plasmon resonance (SPR) sensors: approaching their limits?. Opt. Express.

[21] Bindel, D. & Goodman, J. *Principles of scientific computing* (2009).

[22] Scientific, T. EZ-Link HPDP-Biotin Instructions (2016).

[23] Johnson P, Christy R (1972). Optical constants of the noble metals. Physical Review B.

[24] Whitesides GM, Ostuni E, Takayama S, Jiang X, Ingber D (2001). Soft lithography in biology and biochemistry. Nano Letters.

